# Panel financial ratios data underlying the performance of conventional and islamic banks operating in GCC

**DOI:** 10.1016/j.dib.2019.103979

**Published:** 2019-05-07

**Authors:** Mahieddine A. Ghecham, Abdalla Salih

**Affiliations:** a Al Ain University of Science and Technology, United Arab Emirates; b Abu Dhabi University, United Arab Emirates

## Abstract

This article contains cross-sectional time series (panel) data on 46 banks that operates in Gulf Cooperation Council (GCC) Countries. This data reports a number of financial ratios that are constructed from various financial information listed in the Audited Financial Statements of the banks operating in the GCC countries. The financial statements are available in the official website of the banks. The generated financial ratios were exploited in the published article (Salih, Ghecham and Al Barghouthi, 2018). These financial ratios cover perhaps the largest time span (2006–2012) and the largest number of banks that operate in GCC.

**Table:** Specifications table

Subject area	*Economics and Finance*
More specific subject area	*Economics and Finance*
Type of data	*Table*
How data was acquired	*From Audited Financial Statements listed in the banks' websites.*
Data format	*Raw*
Experimental factors	*Ratios created from mathematical construction of financial indicators of 46 banks divided into 29 Conventional Banks and 17 Islamic banks and which compiles 336 observations.*
Experimental features	*Cross-sectional times series (panel) data covering financial ratios.*
Data source location	*Audited Financial Statement located in the Website of the banks.*
Data accessibility	*Data with this article.*
Related research article	*Salih, A; Ghecham, M.A and Al-Barghouthi, S. (*2018*) “The Impact of Global Financial Crisis on Conventional and Islamic Banks in the GCC countries”.* ***International Journal of Finance and Economics.*** ***In press.***

**Table:** 

**Value of the data** • This data is quite useful in researches that investigate the financial performance of banks. This is relevant for various research communities (academic and professional) who are interested in undertaking comparative studies on Islamic and conventional banks. • This data builds on and extend the scope of existing information on financial ratios of banks that operate in GCC countries. It has, to date, perhaps the largest time span for five different financial performance measures. Therefore, these data can be used as a benchmark for researchers who are interested to build a repository that is endowed with time series information on financial ratios of banks. • This data provides the research community with the opportunity to tap into financial information on two types of banks (Islamic and Conventional Banks). This offers researchers the possibility to undertake valuable comparative studies across GCC region.

## 1 Data

The present data is a product of mathematical transformation of financial indices listed in the Audited Financial Statements of Islamic and Conventional Banks in GCC countries. These statements can be found in a PDF format in the banks’ website. The financial ratios are calculated using established mathematical formula. The calculation process is undertaken in order to generate five different financial ratios (i.e. two profitability ratios namely: Return on Assets and Return on Equity; Efficiency ratio; Liquidity and Solvency ratios) and is replicated for seven consecutive years starting in the year 2006 until 2012. By selecting this period of time, our study chooses large time span that gravitates around the year 2008, the year of the global financial crisis. This means that extending it beyond this period of time to a closer date would not add much to the value of the study in terms of disentangling the difference in performance of the Islamic and conventional banks during the 2008 global financial crisis (see for example, [1], [2], [3].

**Fig. 1:**
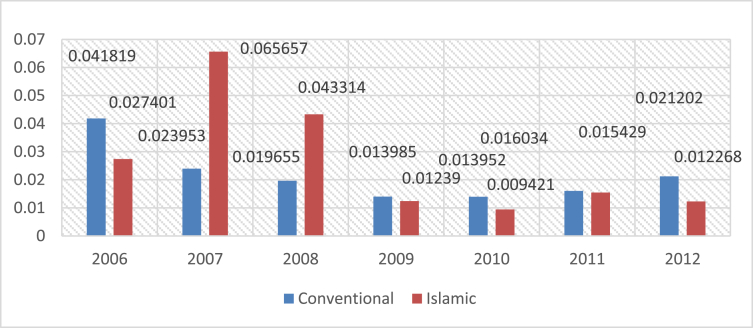
Mean of ROA for islamic and conventional bank between 2006 and 2012.

**Fig. 2:**
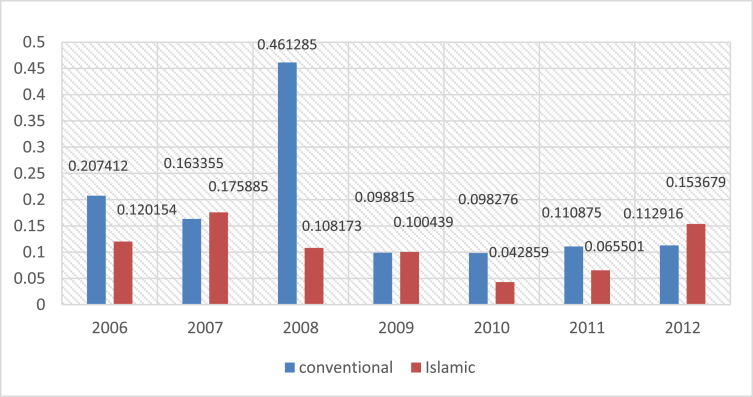
Mean of ROE for islamic and conventional bank between 2006 and 2012.

**Fig. 3:**
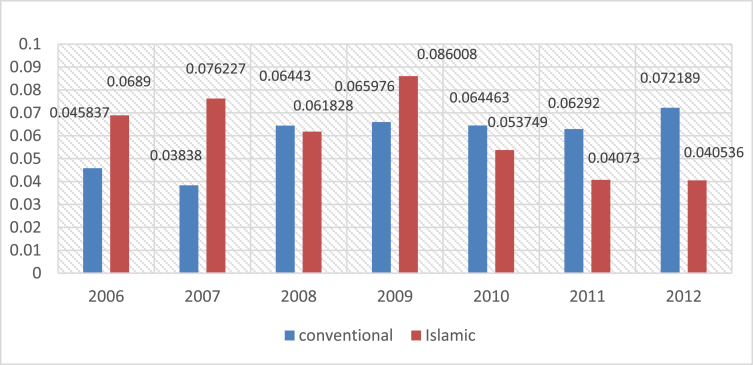
Mean of Efficiency ratio for Islamic and Conventional Bank between 2006 and 2012.

**Fig. 4:**
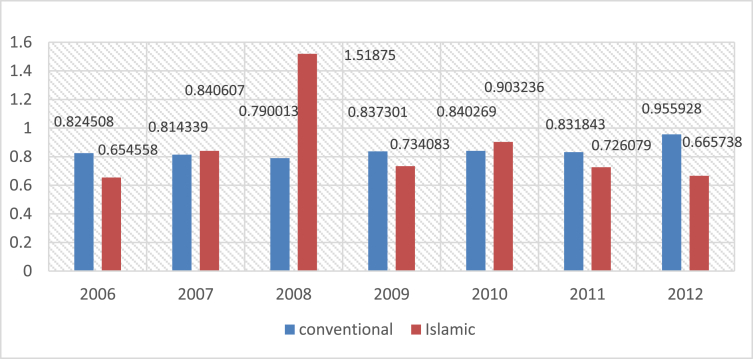
Mean of Liquidity ratio for Islamic and Conventional Bank between 2006 and 2012.

**Fig. 5:**
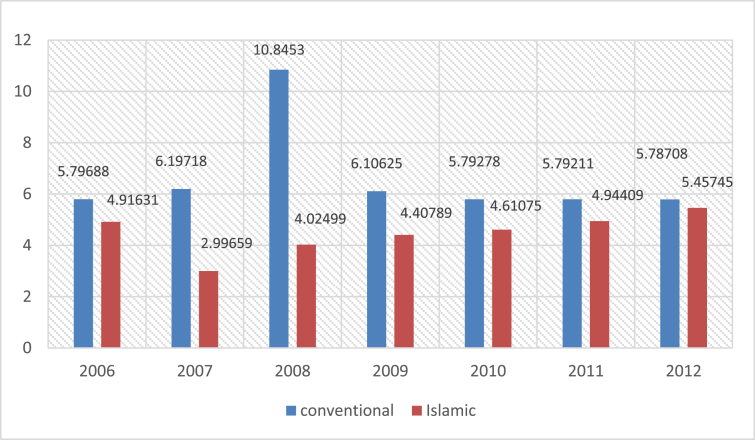
Mean of Solvency ratio for Islamic and Conventional Bank between 2006 and 2012.

The methodology of our paper has concerned 46 banks operating in GCC countries (i.e. United Arab Emirates, Oman, Bahrain, Kuwait, Saudi Arabia and Qatar). The study constructed the financial ratios using the Financial Statements of 29 conventional banks and 17 Islamic banks. (see Fig. 1, Fig. 2, Fig. 3, Fig. 4, Fig. 5).

The following figures provide a descriptive statistics of the mean value of the different constructed financial ratios for the two types of banks.

## 2 Experimental design, materials, and methods

With the use of excel, a number of financial ratios are constructed from audited financial statements of banks. There are 46 banks; these banks have been selected on the basis of their longevity in the market, the size of the geographical area they serve and the common regulations applied to their operations.

The data has been used to construct 5 financial ratios. These are Return On Assets (ROA) ratio, Return On Equity (ROE) ratio, liquidity ratio, efficiency ratio and solvency ratio.

The ROA and ROE are two financial ratios used to gauge the profitability performance of companies.

The ROA gives an idea as to how a company's management is efficient at using assets to generate earnings. It is calculated by dividing the NET INCOME by TOTAL ASSETS (i.e. ROA = Net income/total assets).

The ROE is very useful when it is used to compare the financial performance between similar companies. It is calculated by dividing the NET INCOME by the SHAREHOLDERS′ EQUITY (i.e. ROE = net income/shareholders’ equity).

The liquidity ratio is very important metric that helps appreciate the ability of a company to pay off its debt obligations. It is calculated by dividing TOTAL LOAN by TOTAL Assets (i.e. Liquidity ratio = Total loan/Total assets).

The efficiency ratio is a useful metric as it gives an idea on how successful a company is in using its assets and liabilities internally. It is calculated by dividing TOTAL REVENUE by TOTAL ASSETS (i.e. efficiency ratio = total revenue/total assets).

The solvency ratio is a key metric as it gauges the ability of a company to meets its debt and other financial obligations. It is calculated by dividing DEBT by EQUITY (i.e. solvency ratio = debt/equity).

Our choice of these performance measures stems from our interest in comparing the results of our study with those of prominent studies that used accounting and financial ratios to assess banks performance during the 2008 financial crisis (see for example, [2], [5], [3]; [6]. In fact, our research [4] aimed at compiling important financial and accounting performance measures in one study while considering larger time pan than the one considered by similar studies.
