# Supplementary material for: Panel financial ratios data underlying the performance of conventional and islamic banks operating in GCC
Source: Data Brief. 2019 May 7;24:103979. doi: 10.1016/j.dib.2019.103979 (PMC6536617; doi:10.1016/j.dib.2019.103979)
Supplement: Supplementary file 2 — Multimedia component 2 [file mmc2.docx]

**DATA SOURCE:**

Banks’ financial reports websites addresses as las visited on 30/01/2019:

1. Emirates NBD

https://www.emiratesnbd.com/en/investor-relations/financial-information/annual-reports/

2. Abu Dhabi Commercial Bank

https://www.adcb.com/about/investorrelations/financial-reports/default.aspx

3. Commercial Bank of Dubai

https://www.cbd.ae/corporate/about-cbd/financials-and-investor-relations/annual-report

4. First Gulf Bank

https://www.nbad.com/en-ae/about-nbad/investor-relations/financials/annual-quarterly-reports/FGB-pre-merger-quarterly-and-annual-reports.html

5. Mashreq Bank

https://www.mashreqbank.com/qatar/en/investor-relations/financial-information/annualreports

6. National Bank of Fujairah

https://nbf.ae/en/about-us/investor-relations/financial-information/annual-reports

7. Union National Bank

https://www.unb.com/en/information/about-us/investor-relations/quarterly-results

8. Ajman Bank

http://www.ajmanbank.ae/site/financials.html

9. Bank of Sharjah

https://www.bankofsharjah.com/en/contents/investorlists/financial-statements

10. Emirates Investment Bank

https://www.eibank.com/investor-relations

11. United Arab Bank Consolidated financial statements

https://www.uab.ae/investor-relations/financial-statements

12. The Saudi Investment Bank.

https://saib.com.sa/en/content/financial-reports-2018

13. Samba Financial group

https://www.samba.com/en/about-us/investor-relations/financial-reports.html

14. Ryad Bank

https://www.riyadbank.com/en/about-us/investor-relations/financial-results

15. SAAB Bank

https://www.sabb.com/en/about-sabb/investor-relations/financial-statements/

16. Al Awal Bank

https://www.alawwalbank.com/en/about-us/financial-reports

17. Commercial Bank of Qatar

http://www.cbq.qa/EN/IR/Financial-highlights/Pages/financial-highlights.aspx

18. Doha Bank

http://dohabank.qa/investor/financial-reports/annual-reports/

19. Qatar National Bank https://www.qnb.com/cs/Satellite/QNBQatar/en_QA/InvestorRelations/enFinancialStatement

20. National Bank of Bahrain

https://www.nbbonline.com/en/about-nbb/financial/financial/annual-reports

21. Bahrain Development Bank

http://www.bdb-bh.com/en/financials

22. The Bank of Bahrain and Kuwait https://www.bbkonline.com/InvestorRelations/Financials/Pages/annualreports.aspx

23. Al Ahli Bank of Kuwait

https://abk.eahli.com/abk/InvestorRelations.aspx?id=01

24. Burgan Bank

http://www.burgan.com/aboutus/SitePages/financials.aspx

25. Gulf Bank

https://www.e-gulfbank.com/en/investors/financial-reports/annual-reports

26. Al Tijari Bank commercial bank of Kuwait

https://www.cbk.com/About-CBK/Investors-Relations/Financial-Statements

27. National Bank of Oman

https://www.nbo.om/en/Pages/IR/RESULTS-AND-REPORTS.aspx

28. Bank Musqat

https://www.bankmuscat.com/en/investorrelations/pages/reports.aspx

29. Dubai Islamic Bank

https://www.dib.ae/about-us/investor-relations/financial-information

30. Sharjah Islamic Bank

https://www.sib.ae/Welcome-Investor-Relations/Financial-Information-Investor-Relations#.XDMWnVwzaUk

31. Bank AlBilad

http://www.bankalbilad.com/sites/en/Reports/Pages/fstatement.aspx

32. Bank Aljazira

https://www.baj.com.sa/ar-sa/About-Us/Corporate-Profile/id/142?page=financial-report

33. Alinma Bank https://www.alinma.com/wps/portal/alinma/Alinma/MenuPages/FinancialReports/FinancialStatements/!ut/p/z0/04_Sj9CPykssy0xPLMnMz0vMAfIjo8ziff0tTTy8TQy93c2cXAwcPf0MDEz9_Awt3Ez1g1Pz9AuyHRUBmggHKg!!/

34. Al Rajhi Bank

https://www.alrajhibank.com.sa/en/investor-relations/financials/pages/default.aspx

35. Ithmaar Bank

https://www.ithmaarbank.com/ithmaar-investor-relations/financial_reports

36. Masraf AlRayan

https://www.alrayan.com/english/investor-relations/annual-and-quarterly-financials

37. Barwa Bank

https://www.barwabank.com/investor-relations/financial-information

38. Qatar Islamic Bank

https://www.qib.com.qa/en/InvestorRelations/financial-information/financial-reports.aspx

39. Bahrain Islamic Bank

https://bisb.com/en/who-we-are/financial.html

40. Al Baraka Islamic Bank

https://albaraka.bh/default.asp?action=article&id=54

41. Al Salam Bank

https://www.alsalambahrain.com/en/INVESTOR-RELATIONS/Annual-Reports

42. Khaleeji Commercial Bank

http://www.khcbonline.com/en/InvestorRelations/financial-reports

43. Boubyan Bank

https://boubyan.bankboubyan.com/en/about/reports/

44. Kuwait Finance Bank

https://www.kfh.com/en/home/Personal/Investor-Relations/Financial-Reports.html

45. Bank Dhofar

http://bankdhofar.com/en-GB/Investor_Relations.aspx

46. Ahli Bank

https://ahlibank.om/en-us/investorrelations/Pages/FINANCIAL-REPORTS.aspx
